# Attitudes Towards Animals and Calf Disbudding Techniques: A Mixed Methods Study Using the Animal Attitude Scale (AAS-10)

**DOI:** 10.3390/vetsci12100939

**Published:** 2025-09-28

**Authors:** Andrea D. Calix, Pablo Lamino, Howard Rodríguez-Mori, Arlene Garcia, Elpida Artemiou

**Affiliations:** 1 School of Veterinary Medicine, Texas Tech University, Amarillo, TX 79106, USA; acalix@ttu.edu (A.D.C.); howard.rodriguez-mori@ttu.edu (H.R.-M.); arlene.garcia@ttu.edu (A.G.); 2 Department of Agricultural Education and Communication, University of Florida, Gainesville, FL 32611, USA; pablo.lamino@ufl.edu

**Keywords:** calf disbudding, animal welfare, public perception, caustic paste disbudding, hot-iron disbudding

## Abstract

Calf disbudding is a routine practice in the dairy industry to prevent horn growth and reduce the risk of injury to animals and handlers. However, growing public concern about animal welfare has raised questions about the acceptability of common disbudding methods. This study explored public perceptions of caustic paste and hot-iron disbudding using a mixed methods approach. Quantitative survey analyses captured measurable trends while iterative qualitative analysis explored the underlying reasons behind participant’s attitudes. A convenience sample with a total of 511 Texas resident participants completed a 44-item online survey that included demographic questions, the Animal Attitude Scale (AAS-10), and image-based evaluations of the two techniques. Quantitative analysis using factor analysis and multiple regression revealed that concern for animal welfare and justification for animal use were the most significant predictors (*p* < 0.001) of method acceptability, with caustic paste generally viewed as more humane. Qualitative responses reinforced these results, identifying themes of animal suffering, ethical concerns, and a widespread lack of public knowledge. While caustic paste was preferred, skepticism toward hot-iron disbudding was more pronounced among low-income participants. Nonetheless, when properly performed with pain control, hot-iron disbudding is often considered a more controlled and welfare-conscious method due to faster healing times and reduced risk of injury to other animals from paste exposure. These findings underscore the need for consumer education and transparent communication from the dairy industry.

## Simple Summary

Calf disbudding is a routine practice in the dairy industry to prevent horn growth that later in life may compromise both human and animal safety, yet it often raises public animal welfare concerns. In this study, 511 Texas resident participants evaluated two common techniques for disbudding calves: hot iron and caustic paste, after viewing images and descriptions. This work revealed that most significant predictors of method acceptability were based on concerns for animal welfare and justification for animal use. Overall, 62% of participants perceived caustic paste as more humane, while 28% preferred hot iron when performed with proper pain control. Respondents with higher concern for animal welfare were more accepting of both methods, whereas those supportive of animal use in other contexts were less approving. A notable 43% reported limited knowledge about disbudding. The results also indicated that animal suffering, ethical concerns, and a widespread lack of public knowledge influence how consumers feel about commercial practices that have been identified by the livestock industry as necessary. This work highlights the need for public education and transparent communication from the dairy industry to foster informed opinions and trust.

## 1. Introduction

Disbudding, the removal of horn buds either with caustic paste or hot-iron, is a standard practice in dairy calf management to prevent horn development [1]. Although polled genetics exist (that would prevent the need for disbudding) they are not fully adopted due to availability of bulls with polled genetics, as selective breeding for poses challenges due to the risk of inbreeding and loss of genetic variance [2].

Polled/dehorned cattle are easier to handle and pose a lower risk of injury to other livestock species, farm dogs, as well as humans [3,4]. Polled cattle require less space at the feed bunk in confined housing systems, improving efficiency and reducing competition for resources [5]. From an economic standpoint, polled cattle require less space during transportation, leading to more efficient and cost-effective transport management [6,7].

The two most common disbudding techniques in the dairy industry are caustic paste and hot-iron, both typically performed within the first eight weeks of life [8,9]. Hot-iron disbudding removes the horn bud and cauterizes the tissue to prevent regrowth [10], while caustic paste causes a chemical burn and destroys the developing horn tissue [9]. According to the National Animal Health Monitoring System, among dairy operations that perform disbudding, hot-iron remains the most widely used method, followed by caustic paste (55% and 33% of producers, respectively) [11,12].

An alternative to both techniques is the use of polled genetics, which eliminates the need for disbudding entirely. This approach offers significant long-term welfare benefits by avoiding the pain and risks associated with the procedure, as well as reducing labor and treatment costs [13,14,15]. However, adoption has been slow due to the limited availability of polled dairy sires and concerns over their historically lower genetic merit compared to horned cattle [16,17]. While genetic progress may eventually address these limitations, current industry reliance on disbudding remains high.

Public concern over the ethical implications of animal production systems, including disbudding, has been increasing [18]. Individuals with stronger pro-animal attitudes often show greater empathy toward livestock and higher welfare expectations [18,19]. Dairy farming practices perceived as harmful to animal welfare are met with strong public opposition [20,21], and these concerns influence social license to operate and market trust [22,23,24]. Although pain management during disbudding has improved, understanding public perceptions can help producers make informed decisions, address misconceptions, and align practices with evolving societal expectations [25,26,27,28].

Previous research has shown that consumers often perceive caustic paste as less painful than hot-iron disbudding [29,30]. Building on these findings, the present study aims to: (1) assess public attitudes toward animal welfare using the AAS-10 [31] and examine how these attitudes influence the acceptability of caustic paste versus hot-iron, and (2) explore the reasoning behind the public’s preferred disbudding method.

## 2. Materials and Methods

### 2.1. Ethical Review and Approval

The study was approved by the Human Institutional Research Board (IRB), IRB2024-4 at Texas Tech University. All participants were provided with online written consent before confirming their participation in the study.

### 2.2. Theoretical Framework

The Theory of Cognitive Dissonance guided the analysis to gain participants’ attitudes, perceptions, and decision-making processes. According to Leon Festinger, individuals experience psychological discomfort when their attitudes and behaviors conflict, prompting them to seek resolution [32]. In the context of animal use, individuals may acknowledge potential negative impacts while benefiting from them, creating moral conflict. To reduce discomfort, they may justify actions, such as denying animal consciousness, to reconcile inconsistencies between beliefs and behaviors [33]. For example, while AAS-assessed animal welfare beliefs, the 44-item questionnaire included questions that evaluated dietary choices, purchasing of meat and openness to other alternatives designed to potentially elicit tensions between participants’ values might conflict with their acceptance of farming practices. Furthermore, the inclusion of images of disbudding techniques, aimed to intensify cognitive dissonance and required participants to reconcile welfare beliefs with visible representations of industry norms. The Theory of Cognitive Dissonance is reflected in the selection of predictors (e.g., animal welfare concerns versus justification for animal use) and provided a unified explanation between quantified predictors and qualitative analyses based on open-ended responses.

### 2.3. Questionnaire Development and Participant Recruitment

A 44-item questionnaire was developed in Qualtrics XM (Version April 2024, Qualtrics, Provo, UT, USA) and structured into six sections (see Appendix A).

The first section focused on demographics, asking six questions to better understand the participants’ background. These included age, gender, current city of residence within Texas, educational level, monthly income, and political orientation.

The second section explored participants’ dietary habits through a mix of dichotomous and multiple-choice questions. Topics included whether individuals followed a vegetarian or vegan diet, their openness to purchasing plant-based alternatives, and their actual consumption of such products. Additional questions covered the frequency of meat and dietary consumption, types of meat consumed, and participants’ attitudes toward future behaviors such as purchasing, serving, or consuming beef. This section aims to assess dietary preferences and how they might shape views on animal welfare practices like calf disbudding.

In the third section, participants were asked about their familiarity with the agricultural industry. Questions examined whether they had visited farms or ranches, the types of farms they had visited, their interest in learning about animal agriculture, and their awareness of disbudding practices. This section helped evaluate whether participants’ views on disbudding were based on direct experience, educational exposure, or assumptions.

The fourth section included the validated 10-item Animal Attitude Scale (AAS-10) developed by Herzog et al. (2015) [31], commonly used to assess human attitudes toward animals, including cattle, cats, dogs, and other species [34]. In this study, the AAS-10 measured participants’ views on animal welfare, including ethical concerns about animal use and the balance between opposing animal exploitation and accepting animals for human benefit [35].

In the fifth section, the survey incorporated a standard quality check-question [36] to confirm participant attentiveness. Every one of the 511 respondents answered correctly by selecting “other”, a strong indication of their thoughtful engagement. This question was not included in the final data analysis.

The final section introduced real-life images and definitions of two disbudding techniques: caustic paste (CP) and hot-iron (HI). The selected images were adapted from educational resources used to train farmers and students and approved by an expert in animal behavior to ensure accurate representation of standard procedures. To minimize bias, the descriptions focused on clear and neutral language in definitions and procedural steps described. Participants were provided with standardized descriptions for each: CP was defined as “This (chemical) method is utilized before the horn has attached to the skull, and it is applied as soon as the horn bud can be felt, within the first week of life” [37], and HI as “It is a procedure performed by cauterization using a hot-iron (hot iron disbudding), and it can be carried out when the buds are 5–10 mm long, i.e., generally up to 8 weeks of age” [3]. Participants evaluate the acceptability of both techniques and provided explanations for their choices. Additional questions measured how disbudding might influence their willingness to buy, serve, or consume meat, their interest in learning more about these practices, which disbudding method participants thought the public would prefer, and which disbudding method they felt the industry should adopt, along with justifications. Responses also reflect their views on how scientific evidence might shape public opinion on these procedures.

The survey was administered via Texas Tech University’s Qualtrics system and distributed through Centiment, which recruits participants through online platforms, offers compensation or charitable donation options, and uses IP and cookie-based fingerprinting to prevent duplicates. Recruitment details follow Calix et al. (2025) study [29].

### 2.4. Study Design

While this research employed a non-probabilistic, cross-sectional design with convenience sampling to efficiently reach a broad sample, we acknowledge that Texas-only convenience sampling generalizability to other regions or populations. Cultural and regional attitudes toward animal welfare may not reflect broader U.S. or international perceptions. A mixed-methods sequential explanatory approach was used, collecting and analyzing quantitative data first, followed by qualitative data, to provide both statistical patterns and a deeper context [35].

### 2.5. Quantitative Analysis

Descriptive and inferential analyses were conducted on AAS-10 items and disbudding acceptability measures. Explanatory Factor Analysis (EFA) was used to evaluate the AAS-10 structure. The Kaiser-Meyer-Olkin statistic was 0.815, and Bartlett’s test was significant (X^2^ = 1026.444, *p* < 0.001), confirming suitability for factor analysis. Cronbach’s α assessed internal reliability. Multiple regression analyses examined predictors of acceptability, including demographics and AAS-10 scores. Data was processed in Excel^®^ and analyzed using IBM SPSS Statistics version 29.0.1.1 (171), with statistical significance set at *p* < 0.05.

### 2.6. Qualitative Analysis

Qualitative responses to three open-ended questions in section six were analyzed using Braun and Clarke’s Reflexive Thematic Analysis [38]. Two researchers independently reviewed responses, using entire responses as coding units. Discrepancies were resolved through discussion. Categories were consolidated into themes, illustrated with representative quotes (e.g., animal suffering, lack of knowledge for hot-iron; indecisiveness, supportiveness, and unsupportiveness for both methods).

Throughout the coding process, memos were created to document the rationale behind the creation of the categories. After coding, the researchers then met to discuss and evaluate each category individually, applying reflexivity techniques to ensure that the categories reflected the views of the participants rather than the researchers’ own biases or interpretations. As a result of this iterative process, the researchers realized that saturation was reached halfway through the responses, as no new themes emerged from subsequent categories. After the categories were reviewed, inter-coder reliability was calculated (Cohen’s κ = 0.93, *p* < 0.001).

## 3. Results

Responses from 514 participants were collected, though for analysis purposes, only 511 were included. Two were under the age of 18, and one did not reside in Texas.

### 3.1. Factor Analysis and Reliability Assessment

The EFA yielded two factors with eigenvalues greater than one, which accounted for 33.49 percent and 14.12 percent of the total variance, respectively—results further supported through the scree plot diagram [39].

The two-component solution explained by 47.61 percent of the total variance [40], included extracted factors from the unrotated and rotated models. All subsequent results were based on the rotated factor analysis.

Factor 1, capturing pro-animal use perspectives, demonstrated a Cronbach’s α of 0.73, indicating a good fit. Factor 2, encompassing animal welfare concerns, showed a Cronbach’s α of 0.70, which is considered acceptable. These results suggest that both are reliable and suitable for further analysis, as the Cronbach’s α values fall below commonly accepted thresholds of 0.70 or 0.60 [41].

Additionally, the overall reliability of the AAS-10 survey resulted in a Cronbach’s α of 0.70. While this value meets the higher threshold of the minimally acceptable range (0.60–0.70) [42], it should be interpreted with caution, as it reflects only marginal internal consistency. This suggests that the scale is reasonably reliable for use in this context, but further validation with larger and more diverse samples is recommended to strengthen confidence in its application.

### 3.2. Participant Demographics

The demographic information of participants, including gender, education level, age, and income, was previously documented by Calix et al. (2025) and offers valuable insights into the factors shaping public perceptions of disbudding techniques used in the dairy industry [29].

### 3.3. Quantitative Results: Hot-Iron Acceptability by Demographics and AAS-10 Scores

Multiple regression analyses results examined the acceptability of hot-iron disbudding with predictors including demographic information, AAS-10 Factor 1 (pro-animal use perspectives), and AAS-10 Factor 2 (animal welfare concerns), showed that the regression model met the necessary assumptions, confirming its appropriateness. ANOVA results demonstrated that the model significantly explained variance in acceptability (F (19, 491) = 11.58, *p* < 0.001), indicating that the predictors played a meaningful role in shaping public attitudes.

No autocorrelation was detected, as Durbin-Watson analysis yielded a value of 2.008, indicating that residual correlations were not problematic. Additionally, a multicollinearity check showed that all Variance Inflation Factor (VIF) values were below 3, confirming the absence of severe redundancy among predictors.

The model fit was significant (F (19,491) = 11.58, *p* < 0.001), confirming that the included variables had a meaningful impact on acceptability ratings. The model summary (R^2^ = 0.31, Adjusted R^2^ = 0.28) indicates moderate explanatory power, with the selected predictors accounting for approximately 31.0 percent of the variability in hot-iron disbudding acceptability.

Among the attitudinal predictors, both Justification for Animal Use and Animal Welfare Concerns were statistically significant. For Factor 1, Justification for Animal Use, the results indicated a negative relationship with acceptability (β = −0.326, *p* < 0.001). This suggests that the individuals who support animal use in contexts such as hunting, animal testing, or keeping wild animals in captivity were less likely to find hot-iron disbudding acceptable. Specifically, the acceptability of hot-iron disbudding decreased by 0.326 units for a one-unit increase in justification for animal use (95% CI [−0.453, −0.199]).

In contrast, Factor 2, Animal Welfare Concerns, which reflected attitudes toward using animals in medical research, breeding for skin, and raising cattle for human consumption, had a positive association with acceptability (β = 0.525, *p* < 0.001). This suggests that individuals with stronger animal welfare concerns were more likely to accept hot-iron disbudding. For a one-unit increase in animal welfare concerns, the acceptability of hot-iron disbudding increased by 0.525 units (95% CI [0.398, 0.652]).

Additionally, Knowledge About Animal Welfare was also positively associated with acceptability (β = 0.048, *p* = 0.003), indicating that individuals with greater knowledge of animal welfare were more likely to accept the procedure. For a one-unit increase in knowledge about animal welfare, the acceptability of hot-iron disbudding increased by 0.048 units (95% CI [0.017, 0.079]).

Greater animal welfare concerns and knowledge were linked to higher acceptance of hot-iron disbudding, while greater justification for animal use predicted lower acceptance. These findings also echo into a recurring issue across methods, limited public knowledge, which emerged prominently in the qualitative results.

### 3.4. Demographic Predictors Influencing Hot-Iron Preferences

Neither income nor age groups showed a significant effect on acceptability except for individuals who earn less than $1000 per month, who were slightly less likely to accept hot iron disbudding (β = −0.09, *p* = 0.053). Although this effect was only marginally significant, it suggests that lower-income participants may be less accepting of the procedure compared to those with higher incomes, as shown in Table 1.

### 3.5. Quantitative Results: Caustic Paste Acceptability by Demographics and AAS-10 Scores

The second multiple regression model examining the public’s acceptability of caustic paste disbudding, based on predictors including demographic characteristics (e.g., age, income, education level, gender) and two attitudinal factors: Factor 1 (pro-animal use perspectives) and Factor 2 (animal welfare concerns), which met all assumptions, confirming its appropriateness.

To assess autocorrelation, the Durbin-Watson statistic yielded a value of 2.01, indicating that residuals were not correlated, which supports the validity of the regression results. Additionally, the multicollinearity test confirmed that all Variance Inflation Factor (VIF) values were below 3, meaning that there were no significant redundancies among the predictors, ensuring that each variable contributed independently to the model.

The model fit was significant (F (19,491) = 10.58, *p* < 0.001), confirming that the included variables had a meaningful impact on acceptability ratings. The model summary (R^2^ = 0.29, Adjusted R^2^ = 0.24) indicates moderate explanatory power, with the selected predictors accounting for 29.0 percent of the variability in caustic paste disbudding acceptability.

Among the attitudinal predictors, both Factor 1, Justification for Animal Use, and Factor 2, Animal Welfare Concerns, were statistically significant. Factor 1, Justification for Animal Use, which reflects perspectives on activities such as hunting animals for sport, animal testing, and keeping wild animals in cages, was negatively associated with acceptability for caustic paste disbudding (β = −0.231, *t* = −3.47, *p* < 0.001). This suggests that individuals who support animal use are less likely to accept caustic paste disbudding. Especially, for each one-unit increase in the justification for animal use, the acceptability of caustic paste disbudding decreased by 0.231 units (95% CI [−0.362, −0.100]).

Factor 2, Animal Welfare Concerns, which includes opposition to using animals in medical research, breeding animals for skins, and raising cattle for human consumption, had a positive association with acceptability (β = 0.553, *t* = 8.36, *p* < 0.001), suggesting that individuals with stronger animal welfare concerns were more likely to find caustic paste disbudding acceptable. Therefore, for a one-unit increase in animal welfare concerns, the acceptability of caustic paste increases by 0.553 (95% CI [0.424, 0.682]).

Knowledge about animal welfare was also positively associated with acceptability (β = 0.050, *t* = 3.03, *p* = 0.003), indicating that individuals with greater awareness of animal care practices were more inclined to accept caustic paste disbudding. Specifically, for a one-unit increase in knowledge, the acceptability of caustic paste disbudding increases by 0.050 units (95% CI [0.018, 0.082]).

As with hot-iron, acceptance of caustic paste increased with greater animal welfare concern and knowledge and decreased with greater justification for animal use. This reinforces how the limited public knowledge shapes perceptions of both methods, making it a key theme in understanding acceptability.

### 3.6. Comparison of Predictor Influencing Acceptability of Hot-Iron and Caustic Paste Disbudding

The findings for both hot-iron and caustic paste disbudding techniques indicated considerable similarities, with attitudes toward animal use and animal welfare emerging as the primary factors influencing acceptability. However, age played a notable role in shaping perceptions of caustic paste disbudding, although it did not significantly impact views on hot-iron disbudding. Specifically, middle-aged participants, 35–44 years old, seemed more uncertain about the use of caustic paste, suggesting that generational differences may influence attitudes toward this disbudding technique. Moreover, the model for hot-iron disbudding demonstrated slightly greater explanatory power, indicating that the identified predictors had a stronger influence on their acceptability compared to caustic paste disbudding.

Both multiple regression models satisfied the necessary statistical assumptions, affirming the robustness of the analyses. The ANOVA results for hot-iron disbudding (F (19,491) = 11.58, *p* < 0.001) indicated a marginally higher explanatory power than those for caustic paste disbudding (F (19, 491) = 10.58, *p* < 0.001), reinforcing the assumption that the selected predictors played a more substantial role in shaping public attitudes toward hot-iron disbudding. Regardless of these distinctions, both models consistently demonstrated that demographic variables had a minimal impact on acceptability, while attitudes toward animal welfare and animal use remained the most influential factors.

### 3.7. Demographic Predictors Influencing Caustic Paste Disbudding Preferences

None of the income nor age groups showed a significant effect on acceptability except for those respondents aged 35–44, who were less likely to accept caustic paste disbudding (β = −0.356, *t* = −2.36, *p* = 0.02, 95% CI [−0.655, −0.057]). This finding suggests that middle-aged participants were more skeptical of caustic paste disbudding, potentially due to greater awareness of or exposure to dairy farming practices, differing ethical concerns or generational attitudes toward animal welfare. Generational differences in attitudes toward animal use are well-documented, with younger individuals exhibiting greater moralistic (a strong emphasis on the ethical treatment of animals, advocating against their exploitation and mistreatment) concern for animal welfare, while older individuals, especially farmers and rural residents, tend to adopt a more utilitarian (a primary focus on the functional and economic benefits that animals provide) perspective, which may contribute to varying levels of skepticism towards livestock management practices like disbudding [43] as shown in Table 2.

The results for hot-iron and caustic paste disbudding were largely similar, with attitudes toward animal use and welfare being the strongest factors influencing acceptability for techniques. However, age played a significant role in caustic paste acceptability but not in hot iron disbudding, indicating that middle-aged (35–44 years old) individuals may be more hesitant toward caustic paste. Additionally, the model for hot-iron disbudding had slightly stronger explanatory power, suggesting that the predictors were more influential in determining hot-iron’s acceptability compared to caustic paste.

### 3.8. Qualitative Results: Participants’ Perceptions of Hot-Iron Disbudding

Based on participants’ responses to open-ended questions, the thematic analysis of participants’ perceptions of hot-iron disbudding revealed three primary themes: First, Animal Suffering: This theme includes categories related to animal cruelty, animal welfare, and concerns about the pain associated with the procedure; Second, Practice Endorsement: Participants who were supportive of the practice were categorized under this theme; Third, Opposition to the Practice: This theme includes two categories (opposition to the procedure and religious objections). Fourth, Lack of knowledge consisted of two categories: lack of information and lack of knowledge about hot-iron disbudding, as shown in Table 3.

Often participants’ opinions were influenced by a limited understanding of the procedure. This lack of knowledge was particularly evident when participants were asked about their preferred disbudding technique and their reasoning behind their selection. Educating the public could help align their understanding with current animal welfare practices. The thematic analysis for hot-iron perspectives is summarized in Table 3.

#### 3.8.1. Theme 1: Animal Suffering

More than a third of the participants (n = 192, 37.57%) expressed strong opposition to hot-iron disbudding, perceived it as a cruel and inhumane practice and causing unnecessary suffering to calves and should not be performed. The following categories were derived from participants (P), and the rationale regarding the hot-iron disbudding technique is. Categories described in Table 3.

#### 3.8.2. Theme 2: Practice Endorsement

Several participants (n = 65, 12.72%) expressed support for hot-iron disbudding, viewing it as a humane and safe procedure that is performed for valid reasons. Additionally, some considered it to be less painful compared to caustic paste disbudding. Categories described in Table 3.

#### 3.8.3. Theme 3: Unsupportive of the Hot-Iron Practice

Some participants (n = 56, 10.96%) regarded hot-iron disbudding in dairy calves as unacceptable or entirely unacceptable. Their opposition was based on various concerns, including religious objections, the perception that the procedure was unnecessary, and the belief that it causes significant pain. Additionally, some participants expressed that calves have the right to have their horns, while one participant associated the procedure with animal testing. Categories described in Table 3.

#### 3.8.4. Theme 4: Lack of Knowledge

A number of participants (n = 47, 9.20%) reported being unfamiliar with the hot-iron disbudding procedure, its effects, and the level of pain it may cause in calves. Additionally, participants had questions regarding the procedure. This knowledge gap presents an opportunity to develop educational resources aimed at informing the public and consumers about the technique. Categories described in Table 3.

### 3.9. Qualitative Results: Participants’ Perceptions of Caustic Paste Disbudding

Based on the responses to open-ended questions, the thematic analysis of the participants’ perceptions of caustic paste disbudding identified four key themes. First, Indecisiveness: this theme captures participants mixed feelings and uncertainty regarding the procedure; Second, Lack of Knowledge: regarding both the lack of information and unfamiliarity with caustic paste disbudding; Third, Opposition to Practice: this theme includes sixteen categories, reflecting various concerns such as animal abuse, welfare (both animal and human), pain, ethical objections, and personal discomfort. Specific categories within the themes include disgust, religious objections, unnecessary procedures, and opposition to the practice: Fourth, Support for the Practice, in contrast to the opposition theme, this category represents participants who viewed caustic paste disbudding positively. Categories include perceived humaneness, reduced pain, and support for the procedure, with some expressing conditional approval.

The supportive theme was relevant as it provided a contrast to those who opposed caustic paste disbudding, and participants who favored hot-iron disbudding. This comparison highlights the variability in public perception and the complexity of attitudes toward different disbudding techniques. Table 4 provides a summary of the thematic analysis of perspectives on caustic paste disbudding.

#### 3.9.1. Theme 1: Indecisiveness

A number of participants (n = 58, 11.35%) did not express a clear perspective on the well-being of calves when the caustic paste was used for disbudding. While they generally supported humane practices, they did not provide a definitive perspective or conclusive evidence on the technique. Categories described in Table 4.

#### 3.9.2. Theme 2: Lack of Knowledge

Some participants (n = 69, 13.50%) did not have any prior knowledge about what caustic paste disbudding entailed, so they refused to provide their opinions. They were not sure if this procedure was painful or not, but some showed interest by expressing their need for more information. Categories described in Table 4.

#### 3.9.3. Theme 3: Unsupportive of the Caustic Paste Practice

Almost a third of the participants (n = 161, 31.51%) expressed strong opposition to disbudding, viewing it as a painful, unsafe, and unnecessary procedure performed on calves. Concerns regarding animal cruelty were emphasized in relation to caustic paste disbudding. Many participants also opposed the practice based on religious and ethical grounds, perceiving it as sinful or immoral. Some also expressed that humans should not have the right to inflict pain on calves. Categories described in Table 4.

#### 3.9.4. Theme 4: Supportive of the Practice

A portion of participants (n = 82, 16.05%) generally perceived caustic paste disbudding as an acceptable and favorable technique. Their reasoning suggests that they believe caustic paste may not cause significant pain or harm, including the absence of bleeding in calves. Many participants compared caustic paste to other disbudding techniques, considering it to be a less painful and safer alternative. This perception may have been influenced by the visual representation of caustic paste in the image provided with the survey, which resembles a regular cream, reinforcing the assumption that it is a mild and non—invasive procedure. Categories described in Table 4.

## 4. Discussion

This paper reflects public attitudes and perceptions of Texas residents regarding hot-iron and caustic paste disbudding techniques, surrounding the influence of demographic and attitudinal factors in the acceptability of hot-iron or caustic paste disbudding. The results underline that the attitudes and perceptions of animal welfare, justification of animal use, and ethical concerns were the strongest predictors of acceptability for both methods, with caustic paste being mainly considered more humane than hot-iron disbudding. We acknowledge that attitudes may differ in other U.S. regions with distinct husbandry norms and welfare regulations.

During hot-iron disbudding, the heat cauterizes the blood vessels around the wound causing minimal bleeding but obvious tissue damage, as hot-iron disbudding causes third-degree burns where the hot iron is applied, and first and second-degree burns on the surrounding tissues [44]. It may take seven to nine weeks for calves to fully heal [45,46,47]. Although robust scientific evidence is lacking, caustic paste disbudding has been suggested to be one of the least painful methods for horn bud removal [48]. Caustic paste has been perceived as less painful [49], possibly due to lack of visual damage to the surrounding tissue in the initial days of application. Yet, caustic paste can cause severe wounds and has slower healing times, compared to cautery, based on a limited sample of dairy calves [50] and research in dairy goats [51]. Current research has not focused on further evaluation of caustic paste on dairy calves, such as sensitivity, wound healing and wound size over time [52]. The chemicals used in caustic paste are typically strong alkaline substances like sodium or calcium hydroxide that do not cause immediate pain but instead create third-degree burns that worsen over time [50,52,53]. Studies have shown that healing from caustic paste can take up to 18 weeks, and calves often continue to show signs of discomfort for several weeks after the procedure [50,52]. Conversely, hot-iron disbudding causes immediate pain, but it’s usually done with pain control methods such as nerve blocks or horn bud infiltration and anti-inflammatory drugs [54,55,56]. Other extra-label drugs may be used to mitigate pain (but anal not approved for analgesia associated with dehorning in the United States) [57], as well as homeopathic tinctures such as Dull IT (Dr. Pauls’s Lab, LLC, Mazomaine, WI, USA). The recovery period tends to be shorter, with wounds healing in about 7 to 9 weeks [50]. While both methods involve discomfort, the evidence suggests that hot-iron disbudding is potentially less painful with proper pain management. Practices that reduce long-term pain, improve animal welfare, and educate consumers on these efforts are critical in building trust between the dairy industry and the public. Consumer/public education may promote acceptability of required processing procedures in cattle.

### 4.1. Quantitative Comparison: Predictors of Acceptability for Caustic Paste and Hot-Iron Disbudding

The regression analysis showed that approximately 31.0 percent of the variance in acceptability for hot-iron disbudding (R^2^ = 0.31, Adjusted R^2^ = 0.28) and 29.0 percent for caustic paste disbudding (R^2^ = 0.29, Adjusted R^2^ = 0.24). Attitudes toward animal use and welfare were significant predictors in both models, though their influence varies in strength and direction across the two disbudding methods.

In the hot-iron disbudding model, greater concern for animal welfare was positively associated with acceptability (β = 0.36, *p* < 0.001), indicating that for each one-unit increase in animal welfare concern, acceptability increased by 0.36 units. Conversely, stronger justification for animal use was negatively associated with acceptability (β = −0.22, *p* < 0.001) meaning that individuals who support practices like hunting or animal testing were 0.22 units less likely to find hot-iron disbudding acceptable. Knowledge about animal welfare also had a significant positive effect (β = 0.12, *p* = 0.003), suggesting that individuals who believed having higher animal welfare knowledge were more likely to accept the practice.

In the caustic paste model, animal welfare concern was again a strong predictor (β = 0.41, *p* < 0.001), showing a slightly stronger influence than in the hot-iron model. Justification for animal use maintained a negative relationship (β = −0.19, *p* < 0.001), and knowledge about animal welfare also contributed positively (β = 0.10, *p* = 0.009). These results suggest that concern for animal welfare consistently promotes higher acceptability across both methods, but with a greater effect size for caustic paste. This aligns with Brooks et al. (2021), who noted that caustic paste disbudding has often been marketed as a less painful alternative to hot-iron disbudding [58].

Demographic factors had a minimal effect on acceptability, with gender and education showing no significant impact. However, participants with income levels less than $1000 per month had a decrease in acceptability for hot-iron as a disbudding technique, and participants 35–44 years old were less likely to accept caustic paste as a disbudding technique. This finding suggests that economic factors influence perceptions of dairy management practices, potentially due to differing levels of understanding of dairy industry practices. Kellert [43] noted that younger individuals generally exhibit more moralistic concern for animal welfare, which aligns with the higher acceptability of caustic paste disbudding observed in our study. In contrast, older individuals, particularly those with agricultural backgrounds, may adopt a more utilitarian perspective, which could explain the lower acceptability of both disbudding techniques [43]. These results suggest that perceptions of hot-iron disbudding may have an impact on low-monthly income individuals and perceptions of caustic paste disbudding may vary across generations, both potentially due to differences in exposure and understanding of practices in dairy cattle. In support McKendree et al. (2014) also reported that people’s views on livestock production are shaped by factors like age, income and experience with animal welfare as those who have visited farms or are more familiar with animal welfare issues tend to see these practices differently than those with little or no exposure [59].

### 4.2. Public Perceptions and Thematic Insights from Qualitative Responses

Regression analyses showed that concern for animal welfare, justification for animal use, and knowledge about animal welfare were consistent predictors of acceptability for both hot-iron and caustic paste disbudding. Greater concern for animal welfare and higher knowledge about animal welfare were linked to higher acceptance. Although these patterns were similar across both methods, the effect of animal welfare concern was slightly stronger for caustic paste, aligning with its perception as a less invasive method. Demographic effects were minimal, though lower income was linked to reduced acceptance of hot-iron, and individuals aged 35–44 were less accepting of caustic paste.

Qualitative responses provided depth to these results, revealing that participants who supported the techniques often cited practical or welfare–related reasons, while those opposed referenced ethical, religious, or emotional concerns. Across both methods, a clear and recurring theme was limited public knowledge. Many participants admitted uncertainty about the procedures, suggesting that misconceptions or lack of awareness influenced their judgments. This finding reinforces the quantitative result that higher knowledge levels correspond to greater acceptance.

### 4.3. Mixed Methods Integration

The mixed-methods design helped explain why statistical patterns emerged. For example, the positive link between animal welfare concern and acceptability was reflected in comments describing caustic paste as more humane, even among those with limited knowledge of its application. Conversely, participants with strong justification for animal use often rejected both methods as unnecessary and cruel. These findings show that attitudes toward disbudding are shaped by both values and information gaps, making education an essential approach to building informed public opinions.

### 4.4. Mixed Methods Interpretation

Combining both the quantitative and qualitative findings provided a more comprehensive understanding of how the public perceives calf disbudding. While caustic paste disbudding was generally viewed as the more humane disbudding technique, the public acceptability of the technique was influenced by a variety of factors, such as ethical concerns, unfamiliarity with the process, and demographic variation.

Regarding hot-iron disbudding, the quantitative analysis showed lower acceptability among individuals who supported animal use in contexts such as hunting or animal testing. This was also reflected in the qualitative responses, where several participants expressed discomfort and concern about the pain associated with the procedure. The animal suffering theme, including cruelty and animal welfare concerns categories, was often mentioned by participants who opposed hot-iron disbudding. Participants who supported hot-iron disbudding justified the procedure as being a practical necessity of a traditional farming practice.

Caustic paste disbudding was widely accepted. The quantitative model showed a stronger association between animal welfare concerns and acceptability for caustic paste, compared to hot-iron disbudding, aligning with the qualitative responses, in which participants described caustic paste as causing less pain and being gentler on the animal. Many participants disclosed not entirely understanding how this technique worked, which was reflected in themes related to indecisiveness and lack of knowledge.

By combining both quantitative and qualitative phases, the study highlights that attitudes, knowledge, and ethical reasoning are central to public perceptions of disbudding. Moreover, the qualitative findings help explain variability in the quantitative results, particularly where uncertainty and lack of familiarity influenced participants’ responses. This integration underscores the importance of educational efforts and transparent communication about disbudding practices to align public understanding with current animal welfare standards.

Overall, hot-iron disbudding elicited a stronger negative reaction, whereas caustic paste was considered a moderate procedure that sometimes caused uncertainty among the participants. Participants stated their desire for more information to enhance their knowledge surrounding both practices of disbudding.

### 4.5. Comparison to Previous Research

These findings align with previous studies on public perceptions of farm animal welfare, which have shown consistent opposition to painful livestock procedures [6,20,23,60]. The preference for caustic paste over hot-iron disbudding aligns with existing research evidence indicating that the public tends to favor methods perceived as less invasive [61]. Although caustic paste is increasingly used in the United States and often perceived as less invasive than cautery techniques, it can still cause significant tissue damage, requiring a longer healing time than hot-iron disbudding, and may lead to complications if not applied correctly [62]. Concerns about animal pain and suffering continue to play a crucial role in shaping consumer attitudes toward livestock management. This study expands on prior research by examining the specific role of attitudes toward animal use and welfare in determining perceptions of disbudding [29].

### 4.6. Implications for the Dairy Industry

This study provides valuable insights for dairy farmers and industry stakeholders aiming to address public concerns about disbudding practices. This work can potentially aid dairy farmers and industry stakeholders in developing improved communication strategies, consumer-facing education materials, and possibly even policies that the dairy industry could adopt. Given the strong influence of animal welfare concerns on the acceptability of farm practices, enhancing transparency and public education about pain management strategies is essential to maintain consumer trust. To ensure long-term sustainability, the industry and its stakeholders may need to reevaluate husbandry practices and broader objectives [63]. Farmers and industry professionals may benefit from effective communication initiatives to inform the public about the importance of disbudding and how pain mitigations are implemented. Understanding how demographic and other factors influence public perceptions of dairy cattle production is essential for aligning farm management decisions with consumer expectations [60].

Policy implications from these findings suggest the need for standardized pain management regulations to improve public perception surrounding disbudding. An optimal solution would be to provide education that helps the public better understand modern production practices in livestock operations [22]. Educational initiatives that provide clear, science-based information about disbudding practices can help bridge the gap between industry practices and consumer expectations.

### 4.7. Limitations

This study relied on self-reported data, which may be influenced by social desirability bias. Some limitations include (a) the use of static images instead of video demonstrations, (b) possible response bias from compensation, and (c) limited representativeness of the online survey sample. Additionally, only Texas participants were recruited as well as only through an online survey platform, which may limit representativeness despite efforts to obtain a diverse sample. While the use of images and standardized descriptions aimed to provide clarity, we recognize that images were static instead of video demonstrations and that responses may still have been shaped by individual interpretation or prior exposure to the topic. Lastly, possible response bias from compensation and limited representativeness of the online survey sample may exist.

Although the model was statistically significant, its explanatory power was moderate, accounting for roughly one-third of the variability in acceptability ratings. This indicates that a substantial amount of variation remains unexplained. The study did not capture other potentially influential factors, such as rural versus urban upbringing, prior experience with livestock, or broader cultural exposure to agricultural practices, that may further shape perceptions of hot-iron disbudding [25,26,27,28]. Future research incorporating these variables could provide a more comprehensive understanding of public attitudes.

## 5. Conclusions

Our findings suggest that the most significant factors influencing public acceptance of these procedures are the justification of animal use and attitudes toward animal welfare. Both disbudding techniques were viewed with similar concerns regarding animal suffering; however, caustic paste disbudding was generally perceived as a more humane technique compared to hot-iron disbudding.

Quantitative analysis revealed that demographic factors had a limited influence on acceptability ratings for both disbudding methods, while individual beliefs about animal welfare were the strongest predictors. For caustic paste disbudding, the 35–44-year-old age group was negatively associated with acceptability (β = −0.356, *t* = −2.357, *p* = 0.019, 95% CI [−0.655, −0.057]), indicating that participants in this age range were less likely to find the procedure acceptable compared to the reference group. Income categories did not show significant associations. Among attitudinal factors, justification for animal use was negatively associated with acceptability (β = −0.231, *t* = −3.466, *p* < 0.001, 95% CI [−0.362, −0.100]), meaning participants who support animal use in contexts such as hunting or testing were less likely to accept caustic paste disbudding. Animal welfare concerns were positively associated (β = 0.553, *t* = 8.358, *p* < 0.001, 95% CI [0.424, 0.682]), and Knowledge about animal welfare was also positively associated (β = 0.050, t = 3.035, *p* = 0.003, 95% CI [0.018, 0.082]), indicating that stronger welfare concerns and greater knowledge increased acceptability.

For hot-iron disbudding, the most significant attitudinal predictors followed a similar pattern. Justification for animal use was negatively associated with acceptability (β = −0.326, *t* = −5.010, *p* < 0.001, 95% CI [−0.454, −0.198]), whereas Animal welfare concerns were positively associated (β = 0.525, *t* = 8.117, *p* < 0.001, 95% CI [0.398, 0.652]). Knowledge about animal welfare also showed a positive association (β = 0.048, *t* = 2.988, *p* = 0.003, 95% CI [0.016, 0.080]). Demographic predictors, including age and income, did not show statistically significant effects for hot-iron disbudding. These results highlight the need to improve public education about the procedures, their ethical implications and the pain management strategies used. These results highlight the need to improve public education about the procedures, their ethical implications and the pain management strategies used.

The qualitative analysis underlined the importance of providing more transparent information to the public. A significant portion of respondents expressed mixed feelings or opposition towards both disbudding methods, with several reporting a lack of knowledge as a key factor contributing to their perceptions. Addressing these knowledge gaps through education could help align public understanding with current practices in animal welfare.

In conclusion, the dairy industry should continue to prioritize pain management through the development of strict pain control protocols and work towards providing greater education surrounding disbudding practices to the public through targeted education. Future studies that include longitudinal attitude tracking and intervention trials can further inform the industry. Clear communication and public education are essential for maintaining credibility and ensuring long-term sustainability of the dairy industry. The dairy industry should adopt standardized pain control protocols for all disbudding procedures and implement targeted public education campaigns that explain the rationale, methods, and welfare safeguards in place. These actions can help bridge the gap between public concerns and industry practices, strengthen consumer trust, and support the long-term sustainability of dairy production.

## Figures and Tables

**Table 1:** Regression Analysis for Factors Influencing Hot-Iron Disbudding Preferences.

Model	Unstandardized Coefficients (B)	Std. Error	Standardized Coefficients (Beta)	*t*	Sig.
Intercept	1.611	0.381		4.229	<0.001
Justification Animal Use	−0.326	0.065	−0.217	−5.01	<0.001
Animal Welfare	0.525	0.065	0.357	8.117	<0.001
Level of knowledge about animal welfare	0.048	0.016	0.116	2.988	0.003
Male	0.145	0.095	0.062	1.534	0.126
Incomplete High School	0.217	0.249	0.036	0.873	0.383
Incomplete college/university	0.137	0.119	0.05	1.153	0.249
PhD degree	0.256	0.160	0.066	1.60	0.11
High School	0.148	0.119	0.056	1.25	0.212
$3001–$5000	−0.127	0.126	−0.045	−1.01	0.313
$5001–$7000	−0.065	0.154	−0.018	−0.418	0.676
$7001–$9000	−0.147	0.223	−0.026	−0.66	0.510
$9001–$11,000	−0.295	0.222	−0.053	−1.329	0.184
More than $11,000	−0.054	0.167	−0.014	−0.324	0.746
Less than $1000	−0.283	0.146	−0.089	−1.939	0.053
18–24 years old	−0.110	0.168	−0.031	−0.653	0.514
25–34 years old	−0.223	0.150	−0.068	−1.483	0.139
35–44 years old	−0.235	0.147	−0.073	−1.594	0.112
45–54 years old	0.059	0.151	0.018	0.393	0.694
55–64 years old	0.027	0.139	0.009	0.195	0.845

**Table 2:** Regression Analysis for Factors Influencing Caustic Paste Disbudding Preferences.

Model	Unstandardized Coefficients (B)	Std. Error	Standardized Coefficients (Beta)	*t*	Sig.
Intercept	1.618	0.390		4.147	<0.001
Justification for Animal Use	−0.231	0.067	−0.152	−3.466	<0.001
Animal Welfare	0.553	0.066	0.373	8.358	<0.001
Level of knowledge about animal welfare	0.050	0.016	0.119	3.035	0.003
Male	0.123	0.097	0.052	1.266	0.206
Incomplete High School	−0.254	0.255	−0.041	−0.997	0.319
Incomplete college/university	0.110	0.122	0.040	0.908	0.365
PhD degree	0.215	0.164	0.055	1.311	0.191
High School	0.046	0.121	0.017	0.377	0.706
$3001–$5000	−0.149	0.129	−0.052	−1.162	0.246
$5001–$7000	−0.055	0.158	−0.015	−0.35	0.727
$7001–$9000	−0.001	0.229	0	−0.005	0.996
$9001–$11,000	−0.296	0.228	−0.053	−1.300	0.194
More than $11,000	−0.174	0.171	−0.043	−1.021	0.308
Less than $1000	−0.261	0.15	−0.081	−1.748	0.081
18–24 years old	−0.018	0.172	−0.005	−0.107	0.915
25–34 years old	−0.247	0.154	−0.075	−1.607	0.109
35–44 years old	−0.356	0.151	−0.109	−2.357	0.019
45–54 years old	0.042	0.155	0.012	0.274	0.784
55–64 years old	0.045	0.142	0.015	0.316	0.752

**Table 3:** Participants’ Hot Iron Perceptions.

Themes	Categories	Participant’s Rationale (P = Participant)
Animal suffering	Animal cruelty	“It’s animal abuse/cruelty” (P43). “This seems unnecessarily cruel” (P88).
	Animal welfare concerns	“The process appears to stress the animal, and it doesn’t look happy” (P311). “Does not look humane” (P388).
	Concerns about pain	“Looks painful” (P296). “It looks extremely painful for the cows. If they’re going to be raised for food anyway, they should live pain-free lives first” (P444).
Practice endorsement	Supportive of the practice	“This is done for a reason and does not hurt the animal”(P6). “It is done early in life to have the least amount of pain inflicted” (P379)
Unsupportive of the practice	Opposition to the practice	“It looks and sounds horrible”(P119). “I do not support altering the animal” (P423).
	Religious objections	“They are living beings that feel pain. God created them with horns” (P210). “Jehovah’s do not believe in torturing animals”(P469).
Lack of knowledge	Lack of information	“Not enough information given to determine how painful the procedure is” (P491).
	Lack of knowledge	“This is the first time I have heard of this procedure” (P31). “Not knowledgeable about this procedure” (P84).

**Table 4:** Participants’ Caustic Paste Perceptions.

Themes	Categories	Participant’s Rationale (P = Participant)
Indecisiveness	Mixed feelings	“I don’t know how I feel about it” (P425). “It’s not very bad but not good either” (P438).
	Uncertainty	“I’m not sure if this is cruel or not” (P122). “Cannot tell if animal is in unnecessary pain” (P289).
Lack of Knowledge	Lack of information	“This doesn’t explain if it harms the animal” (P264). “I need more information other than pictures” (P430).
	Lack of knowledge	“I do not know enough about process” (P101). “I’m not knowledgeable on the subject” (P163).
Unsupportive of the Practice	Animal abuse	“That would be considered abuse” (P204). “That is totally abuse of an animal” (P313).
	Animal cruelty	“It looks cruel” (P89). “I think it is cruel and there is no need to do it” (P162).
	Animal welfare and human concerns	“I don’t like nobody, human or animal, to be hurt” (P58).
	Animal welfare concerns	“No animal should have to endure pain” (P152). “It looks like it hurts them” (P429).
	Concerns about pain	“It looks painful and burning for the animals” (P170). “Just the image looks painful” (P460).
	Disgust and inhumane practice	“This is gross and inhumane” (P86).
	Ethical objections	“I feel it is wrong for humans to chemically modify animals” (P414). “Harm being done to animals for some type of gain is unethical” (P509).
	Inhumane and unethical	“It’s inhumane and unethical. They were born with it shouldn’t be removed” (P194).
	Inhumane practice	“I see no reason for it, inhumane, unnatural” (P125). “It’s inhumane and unnecessary” (P408).
	Not necessary	“I don’t feel it is necessary to remove their horns” (P397)
	Opposition to the practice	“Not good at all” (P53). “Doesn’t seem right” (P381).
	Personal dislike	“Didn’t like it” (P161).
	Religious objections	“No one has the right to tamper with Jesus Christ’s creation” (P17). “They are meant to have horns because God created them that way” (P210).
	Uncomfortable practice	“It makes me uncomfortable that something was removed from their body” (P94)
	Unnecessary	“I don’t think it’s necessary and it’s cruel” (P99).
	Unsupportive of the practice	“It’s ridiculous to do this to animals” (P93) “I don’t endorse doing this to any animals” (P110).
Supportive of the practice	Good reason	“There is good reason for them to do this” (P352).
	Harmless procedure	“I see no harm” (P494).
	Humane practice	“Does not harm the stock, very humane” (P69). “Seems humane” (P401).
	Less painful	“It appears less invasive” (P105). “It’s the easiest and less painful” (P130).
	Painless procedure	“It appears to be painless” (P396).
	Supportive of the practice	“It stops the growth before the animal knows what is happening” (P57). “It is removed before it completely attaches to the skull so might be better” (P256).
	Supportive of the practice with caution	“The reasons for this practice are valid, but proper pain management should be serious consideration for this procedure” (P363). “I believe the method should only be used based on the pain inflicted on the animal” (P491).

## Data Availability

The original contributions presented in this study are included in the article. Further inquiries can be directed to the corresponding author(s).

## Appendix A. Questionnaire for the Public

**Consent Form**	

**Exploring the Calf Disbudding Procedures Perceptions in Texas**	
Thank you for participating in this study!	
The survey is designed to understand your perception of calf disbudding	
- Your participation is completely voluntary.	
- You can skip any question or stop at any point.	
- Your response will be completely anonymous.	
- The estimated duration is 10 min.	

**Do you agree to participate?**	
⬜ Yes	
⬜ No	
⬜ I would like to have more information	

**Exploring the Calf Disbudding Procedures Perceptions in Texas**	

**What is this research studying?**	
This study will help us learn how people involved and perform disbudding procedures perceive the methods utilized on cow—calf operations. Specifically, we are interested in people’s views surrounding the effect of caustic paste or hot—iron on animal behavior and welfare.	

**What would I do if I participated?**	
In this study, you will be asked to complete a questionnaire to share your thoughts about your experience(s) and view(s) with disbudding procedures to destroy horn-producing cells in young calves. You will also view two images that show the two disbudding procedures of caustic paste and hot—iron and ask for your opinions. The questions and images may make you feel uncomfortable. Your participation is voluntary, and you are free to withdraw from this study at any time. Should you decide to withdraw from the study, your responses will be deleted. There will be no compensation for participating in the study.	

**Can I quit if I become uncomfortable?**	
Yes, absolutely, Dr. Artemiou and Texas Tech University’s Institutional Review Board have reviewed this research project and think you can participate comfortably. However, you can skip parts of the research you are not comfortable with and stop at any time. You will keep all the benefits of participating even if you stop. Participating is your choice.	

**How long will participation take?**	
We are asking for 10 min of your time.	

**How are you protecting privacy?**	
Your name will not be collected not linked to any material in reports, publications, or presentations. No one rather than the researchers associated with this project will have access to the raw data. All related documentation will be stored in the researcher’s locked office and on a password protected computer.	

**What will happen to my data?**	
Your information collected as part of the research, even if identifiers are removed, will not be used, or distributed for future research studies.	

**What are the benefits and risks of participating in this research?**	
It is anticipated that there is only minimal risk associated with participation in this study. Minimal risk means that the risks of physical, psychological, or social harm posed are not greater than those ordinarily encountered in daily life. You may experience some time restrictions associated with completing the questionnaire, and to minimize it we will ensure to offer flexibility and accommodate your schedules. Also, some of the questions and the videos may make you feel uncomfortable when viewing and/or sharing information surrounding disbudding techniques may be experienced, and we will ensure confidentiality to your responses.	
The benefits associated with this research will be gain better understanding surrounding the different views regarding the two disbudding procedures and ultimately inform recommendations to improve animal health and welfare.	
We appreciate your time and effort with this research study.	

**I have some questions about this study. Who can I ask?**	
The study is being run by Dr. Artemiou and Dr. Garcia from the Department of School of Veterinary Medicine at Texas Tech University. If you have questions, you can call them at 806-742-2011. Texas Tech University also has a Board that protects the rights of people who participate in research. You can contact them at 806-742-2064 or hrpp@ttu.edu	

**Do you agree to participate?**	
⬜ Yes	
⬜ No	

**Demographic Information**	

**Age:**	
⬜ Less than 18 years old.	
⬜ 18–24 years old.	
⬜ 25–34 years old.	
⬜ 35–44 years old.	
⬜ 45–54 years old.	
⬜ 55–64 years old.	
⬜ 65 years old or more.	

Gender	
⬜ Female	
⬜ Male	
⬜ Other	
⬜ Prefer not to say	

City in TX you are currently residing at _________________.	

Highest Level of Education	
⬜ Incomplete Elementary School	
⬜ Elementary School	
⬜ Incomplete High School	
⬜ High School	
⬜ Incomplete College/University	
⬜ College/University	
⬜ Master’s degree	
⬜ Ph.D. degree	

Monthly Income	
⬜ Less than $1000	
⬜ $1000–$3000	
⬜ $3001–$5000	
⬜ $5001–$7000	
⬜ $7001–$9000	
⬜ $9001–$11,000	
⬜ More than $11,000	

Political Beliefs	
⬜ Liberal	
⬜ Very Liberal	
⬜ Neutral	
⬜ Conservative	
⬜ Very Conservative	

**Dietary Habits**	
For the following, consider your daily dietary habits:	

In a week, how many times do you consume meat products?	
⬜ More than two times per day	
⬜ Two times per day	
⬜ One time per day	
⬜ Everyday	
⬜ Every other day	
⬜ Three times per week	
⬜ Less than three times per week	

In a week, what kind of meat products do you consume?	
⬜ Beef	
⬜ Pork	
⬜ Poultry	
⬜ Lamb	
⬜ Veal	
⬜ Seafood	
⬜ Game Meat (deer, elk, bison, boar)	
⬜ Specialty Meat (ostrich, emu, alligator, goat, rattlesnake)	

	**Strongly Agree**	**Somewhat Disagree**	**Neither Agree Nor Disagree**	**Somewhat Agree**	**Strongly Agree**
I would purchase beef meat	⬜	⬜	⬜	⬜	⬜
I would consume beef meat	⬜	⬜	⬜	⬜	⬜
I would serve beef meat to my loved ones.	⬜	⬜	⬜	⬜	⬜

Please indicate your level of agreement with the following statement about your future behavior toward beef meat.

Please specify what dairy products do you consume
⬜ Milk
⬜ Cheese
⬜ Yogurt
⬜ Butter
⬜ Ice Cream
⬜ Cream
⬜ Cream Cheese
⬜ Sour Cream

**Animal Attitude Scale**

Instructions: Listed below are a series of statements regarding the use of animals. Circle the letters that indicate the extent to which you agree or disagree with the statement:

SD = Strongly Disagree (1), D = Disagree (2), U = Undecided (3), A = Agree (4), SA = Strongly Agree (5)

	**Statement**	**SD**	**D**	**U**	**A**	**SA**
A1	It is morally wrong to hunt wild animals just for sport.					
A2	I do not think that there is anything wrong with using animals in medical research.					
A3	I think it is perfectly acceptable for cattle and hogs to be raised for human consumption.					
A4	Basically, humans have the right to use animals as we see fit.					
A5	The slaughter of whales and dolphins should be immediately stopped even if it means some people will be put out of work.					
A6	I sometimes get upset when I see wild animals in cages at zoos.					
A7	Breeding animals for their skins is a legitimate use of animals.					
A8	Some aspects of biology can only be learned through dissecting preserved animals such as cats.					
A9	It is unethical to breed purebred dogs for pets when millions of dogs are killed in animal shelters each year.					
A10	The use of animals such as rabbits for testing the safety of cosmetics and household products is unnecessary and should be stopped.					

**Quality Check**
People vary in the amount they pay attention to these kinds of surveys. If you read these questions carefully, please select “other”.

⬜ 1
⬜ 2
⬜ 3
⬜ 4
⬜ 5
⬜ Other

**Images Questions: Technique**
Disbudding with caustic paste images:

For this research, we will use the following definition for caustic-paste disbudding:

“This (chemical) method is utilized before the horn has attached to the skull, and it is applied as soon as the horn bud can be felt, within the first week of life” [37].

Please watch the images and rate how acceptable this disbudding method is for you:

**Mark Your Level Acceptability**				
Totally unacceptable	Unacceptable	Neutral	Acceptable	Totally acceptable
⬜	⬜	⬜	⬜	⬜

Please explain your rationale regarding your answer to the previous question.
_____________________________________________________________________

Please indicate your level of agreement with the following statements about your future behavior toward beef meat that include the disbudding procedure you just viewed.

	**Strongly Disagree**	**Somewhat Disagree**	**Neither Agree Nor Disagree**	**Somewhat Agree**	**Strongly Agree**
I would purchase beef meat that includes the disbudding procedure I just viewed.	⬜	⬜	⬜	⬜	⬜
I would consume beef meat that includes the disbudding procedure I just viewed.	⬜	⬜	⬜	⬜	⬜
I would serve beef meat that includes the disbudding procedure I just viewed to my loved ones.	⬜	⬜	⬜	⬜	⬜

Disbudding using hot iron images.
For this research, we will use the following definition for hot iron disbudding:

“It is a procedure performed by cauterization using a hot iron (hot iron disbudding), and it can be carried out when the buds are 5–10 mm long, i.e., generally up to 8 weeks of age” [62].

Please watch the images and rate how acceptable this disbudding method is for you.

**Mark Your Level Acceptability**				
Totally unacceptable	Unacceptable	Neutral	Acceptable	Totally acceptable
⬜	⬜	⬜	⬜	⬜

Please explain your rationale regarding your answer to the previous question.
_____________________________________________________________________

Please indicate your level of agreement with the following statements about your future behavior toward beef meat that include the disbudding procedure you just viewed.

	**Strongly Disagree**	**Somewhat Disagree**	**Neither Agree Nor Disagree**	**Somewhat Agree**	**Strongly Agree**
I would purchase beef meat that includes the disbudding procedure I just viewed.	⬜	⬜	⬜	⬜	⬜
I would consume beef meat that includes the disbudding procedure I just viewed.	⬜	⬜	⬜	⬜	⬜
I would serve beef meat that includes the disbudding procedure I just viewed to my loved ones.	⬜	⬜	⬜	⬜	⬜

Which method do you prefer to be used in the dairy industry?
⬜ Caustic paste
⬜ Hot iron

Please share your reasoning behind the disbudding method you chose as most preferable.
______________________________________________________________________________

Which disbudding method do you believe the public would prefer?
⬜ Caustic paste
⬜ Hot iron

Will it change your mind if you were provided scientific evidence that one disbudding method is more humane than the other?
⬜ Yes
⬜ No

## References

[1] Byrd C., Craig B., Eicher S., Radcliffe J., Lay D. (2019). Assessment of disbudding pain in dairy calves using nonlinear measures of heart rate variability. J. Dairy Sci..

[2] Grobler R., van Marle-Köster E., Visser C. (2021). Challenges in selection and breeding of polled and scur phenotypes in beef cattle. Livest. Sci..

[3] Stafford K., Mellor D. (2005). Dehorning and disbudding distress and its alleviation in calves. Vet. J..

[4] Yost C. (2023). Dehorning: What Are Your Options?. https://extension.psu.edu/dehorning-what-are-your-options.

[5] Marquette G.A., Ronan S., Earley B. (2023). Calf disbudding—Animal welfare considerations. J. Appl. Anim. Res..

[6] Placzek M., Christoph-Schulz I., Barth K. (2021). Public attitude towards cow-calf separation and other common practices of calf rearing in dairy farming—A review. Org. Agric..

[7] Faulkner P., Weary D. (2000). Reducing pain after dehorning in dairy calves. J. Dairy Sci..

[8] Vickers K., Niel L., Kiehlbauch L., Weary D. (2005). Calf response to caustic paste and hot-iron dehorning using sedation with and without local anesthetic. J. Dairy Sci..

[9] Ede T., von Keyserlingk M.A., Weary D.M. (2020). Conditioned place aversion of caustic paste and hot-iron disbudding in dairy calves. J. Dairy Sci..

[10] Colston K.P., Ede T., Mendl M.T., Lecorps B. (2024). Cold therapy and pain relief after hot-iron disbudding in dairy calves. PLoS ONE.

[11] Sheedy D.B., Aly S.S., Tucker C.B., Lehenbauer T.W. (2024). Mini-review: The history and future of the cornual nerve block for calf disbudding. JDS Commun..

[12] United States Department of Agriculture AaPHIS (2016). Dairy Cattle Management Practices in the United States, 2014. https://www.aphis.usda.gov/sites/default/files/dairy14_dr_parti_1.pdf.

[13] Hanson M. (2021). The Fast Track to Polled Genetics: Dairy Herd Management. https://www.dairyherd.com/news/education/fast-track-polled-genetics.

[14] Thompson N.M., Widmar N.O., Schutz M.M., Cole J.B., Wolf C.A. (2017). Economic considerations of breeding for polled dairy cows versus dehorning in the United States. J. Dairy Sci..

[15] Hill A. (2015). Polled Genetics: Cornell Applied Dairy Cattle Genetics. https://blogs.cornell.edu/dairygenetics/education/polled-genetics/.

[16] Mueller M., Cole J., Sonstegard T., Van Eenennaam A. (2019). Comparison of gene editing versus conventional breeding to introgress the POLLED allele into the US dairy cattle population. J. Dairy Sci..

[17] (2025). No Compromising with Today’s Polled Genetics: ABS Global US. https://www.absglobal.com/no-compromising-polled-genetics/.

[18] Alonso M.E., González-Montaña J.R., Lomillos J.M. (2020). Consumers’ concerns and perceptions of farm animal welfare. Animals.

[19] Cornish A., Raubenheimer D., McGreevy P. (2016). What we know about the public’s level of concern for farm animal welfare in food production in developed countries. Animals.

[20] Ly L.H., Ryan E.B., Weary D.M. (2021). Public attitudes toward dairy farm practices and technology related to milk production. PLoS ONE.

[21] Sirovica L., Ritter C., Hendricks J., Weary D., Gulati S., Von Keyserlingk M. (2022). Public attitude toward and perceptions of dairy cattle welfare in cow-calf management systems differing in type of social and maternal contact. J. Dairy Sci..

[22] Wolf C., Tonsor G., McKendree M., Thomson D., Swanson J. (2016). Public and farmer perceptions of dairy cattle welfare in the United States. J. Dairy Sci..

[23] Robbins J., Proudfoot K., Strand E., Hemsworth L., Coleman G., Hemsworth P., Skuse J., Krawczel P., Van Os J. (2024). Perceptions of dairy cow–handling situations: A comparison of public and industry samples. J. Dairy Sci..

[24] Saraceni J., Renaud D.L., Nelson E., Van Os J.M., Miltenburg C., Winder C.B. (2022). Ontario dairy producers’ perceived barriers and motivations to the use of pain control for disbudding and dehorning calves: A qualitative study. Animals.

[25] Jackson A., Doidge C., Green M., Kaler J. (2022). Understanding public preferences for different dairy farming systems using a mixed-methods approach. J. Dairy Sci..

[26] De la Fuente M.F., Souto A., Caselli C., Schiel N. (2017). People’s perception on animal welfare: Why does it matter?. Ethnobiol. Conserv..

[27] Clark B., Stewart G.B., Panzone L.A., Kyriazakis I., Frewer L.J. (2016). A systematic review of public attitudes, perceptions and behaviours towards production diseases associated with farm animal welfare. J. Agric. Environ. Ethics.

[28] Weary D., Von Keyserlingk M. (2017). Public concerns about dairy-cow welfare: How should the industry respond?. Anim. Prod. Sci..

[29] Calix A.D., Lamino P., Rodríguez-Mori H., Garcia A., Artemiou E. (2025). Public Perceptions of Calf Disbudding Techniques Used on Texas Farms. Animals.

[30] Saraceni J., Winder C.B., Renaud D.L., Miltenburg C., Nelson E., Van Os J.M. (2021). Disbudding and dehorning practices for preweaned dairy calves by farmers in Wisconsin, USA. J. Dairy Sci..

[31] Herzog H., Grayson S., McCord D. (2015). Brief measures of the animal attitude scale. Anthrozoös.

[32] Asmundson G.J.G. (2022). Comprehensive Clinical Psychology.

[33] Knight S., Barnett L. (2008). Justifying attitudes toward animal use: A qualitative study of people’s views and beliefs. Anthrozoös.

[34] Bradley A., Mennie N., Bibby P.A., Cassaday H.J. (2020). Some animals are more equal than others: Validation of a new scale to measure how attitudes to animals depend on species and human purpose of use. PLoS ONE.

[35] Sleegers W. (2024). Understanding People’s Attitudes Towards Wild Animal Welfare: Faunalytics. https://faunalytics.org/understanding-peoples-attitudes-towards-wild-animal-welfare/.

[36] Kung F.Y., Kwok N., Brown D.J. (2018). Are attention check questions a threat to scale validity?. Appl. Psychol..

[37] Bechtel W. (2018). Disbudding with Caustic Paste: Dairy Herd Management. https://www.dairyherd.com/news/disbudding-caustic-paste.

[38] Braun V., Clarke V. (2006). Using thematic analysis in psychology. Qual. Res. Psychol..

[39] Cattell R. (1966). The screen test for the number of factors. Multivar. Behav. Res..

[40] Pett M.A., Lackey N.R., Sullivan J.J. (2003). Making Sense of Factor Analysis: The Use of Factor Analysis for Instrument Development in Health Care Research.

[41] Van Griethuijsen R.A., van Eijck M.W., Haste H., Den Brok P.J., Skinner N.C., Mansour N., Gencer A.S., BouJaoude S. (2015). Global patterns in students’ views of science and interest in science. Res. Sci. Educ..

[42] Ab Hamid M.R., Sami W., Sidek M.M. (2017). Discriminant Validity Assessment: Use of Fornell & Larcker Criterion Versus HTMT Criterion.

[43] Kellert S.R. (1984). American Attitudes Toward and Knowledge of Animals: An Update.

[44] Taschke A.C., Fölsch D.W. (1997). Ethological, physiological and histological aspects of pain and stress in cattle when being dehorned. Tierarztl Prax.

[45] Adcock S.J.J., Tucker C.B. (2018). The effect of disbudding age on healing and pain sensitivity in dairy calves. J. Dairy Sci..

[46] Adcock S.J.J., Vieira S.K., Alvarez L., Tucker C.B. (2019). Iron and laterality effects on healing of cautery disbudding wounds in dairy calves. J. Dairy Sci..

[47] Reedman C.N., Duffield T.F., DeVries T.J., Lissemore K.D., Adcock S.J., Tucker C.B., Parsons S.D., Winder C.B. (2022). Effect of plane of nutrition and analgesic drug treatment on wound healing and pain following cautery disbudding in preweaning dairy calves. J. Dairy Sci..

[48] Vasseur E., Borderas F., Cue R.I., Lefebvre D., Pellerin D., Rushen J., Wade K., de Passillé A. (2010). A survey of dairy calf management practices in Canada that affect animal welfare. J. Dairy Sci..

[49] United States Department of Agriculture AaPHIS (2018). Dairy 2014: Health and Management Practices on US Dairy Operations, 2014.

[50] Lindén J., Taponen S., Talvitie V., Leppävuori E., Hänninen L. (2023). Histopathological findings in a pilot study of dairy calves disbudded with hot cauterization or caustic paste. J. Comp. Pathol..

[51] Hempstead M.N., Waas J.R., Stewart M., Cave V.M., Sutherland M.A. (2018). Evaluation of alternatives to cautery disbudding of dairy goat kids using physiological measures of immediate and longer-term pain. J. Dairy Sci..

[52] Drwencke A.M., Adcock S.J.J., Tucker C.B. (2023). Wound healing and pain sensitivity following caustic paste disbudding in dairy calves. J. Dairy Sci..

[53] Stock M.L., Baldridge S.L., Griffin D., Coetzee J.F. (2013). Bovine Dehorning: Assessing Pain and Providing Analgesic Management. Vet. Clin. N. Am. Food Anim. Pract..

[54] Casoni D., Mirra A., Suter M.R., Gutzwiller A., Spadavecchia C. (2019). Can disbudding of calves (one versus four weeks of age) induce chronic pain?. Physiol. Behav..

[55] Calderón-Amor J., Gallo C. (2020). Dairy calf welfare and factors associated with diarrhea and respiratory disease among Chilean dairy farms. Animals.

[56] Ollivett T. (2019). All dehorning should include pain control. Hoard’s Dairyman.

[57] American Association of Bovine Practitioners (2019). Dehorning Guidelines.

[58] Still Brooks K.M., Hempstead M.N., Anderson J.L., Parsons R.L., Sutherland M.A., Plummer P.J., Millman S.T. (2021). Characterization of efficacy and animal safety across four caprine disbudding methodologies. Animals.

[59] McKendree M.G., Croney C.C., Widmar N.O. (2014). Effects of demographic factors and information sources on United States consumer perceptions of animal welfare. J. Anim. Sci..

[60] Widmar N.O., Morgan C.J., Wolf C.A., Yeager E.A., Dominick S., Croney C.C. (2017). US resident perceptions of dairy cattle management practices. Agric. Sci..

[61] Saraceni J. (2021). The Use of Pain Control for Disbudding and Dehorning Dairy Calves by Farmers in Wisconsin, USA and Ontario, Canada.

[62] Stafford K.J., Mellor D.J. (2011). Addressing the pain associated with disbudding and dehorning in cattle. Appl. Anim. Behav. Sci..

[63] Cardoso C.S., Von Keyserlingk M.A., Hötzel M.J. (2017). Brazilian citizens: Expectations regarding dairy cattle welfare and awareness of contentious practices. Animals.

